# Developmental Features of Lexical Richness in English Writings by Chinese L3 Beginner Learners

**DOI:** 10.3389/fpsyg.2021.752950

**Published:** 2021-09-24

**Authors:** Xuelan Li, Huiping Zhang

**Affiliations:** School of Foreign Languages, Northeast Normal University, Changchun, China

**Keywords:** developmental features, lexical richness, L3 writings, Chinese L3 beginner learners, dynamic usage-based approach

## Abstract

Lexical richness is considered as one of the most efficient methods for assessing writing proficiency and development. However, the developmental features of lexical richness in L3 writing remain relatively poorly understood compared with that of L2 writings. This study reports a cross-sectional corpus-based study that aims to explore the developmental features of lexical richness in L3 writings by Chinese beginner learners of English from the perspective of the dynamic usage-based approach. Specifically, this study compares samples of English writing by Chinese L3 secondary students (grades 7–12) aged 13–18 across three learning stages in terms of lexical sophistication, lexical diversity and lexical density. The writing samples were collected from the Writing Corpus of Chinese Ethnic Minority Beginner Learners as the Third Language (WCCMBL), and the sample sizes of the three stages remained almost the same. The results revealed that lexical richness was generally low in L3 beginner learners' writing. Specifically, L3 beginner learners used fewer diverse words and lexical words, but used numerous high-frequency words in their writing. Additionally, lexical sophistication and lexical density yielded positive growth across the three learning stages, whereas lexical diversity developed non-linearly. These findings reveal a dynamic development of lexical richness in L3 writings, with each of the three measures developing unevenly. Drawing upon these findings, several suggestions for L3 vocabulary teaching are also proposed.

## Introduction

Third language acquisition research has progressed rapidly in the last four decades, and it has recently been recognized as a component of second language acquisition (Sharwood Smith, 1995; Larsen-Freeman and Long, 2000; Ellis, 2013). However, with the spread of English as the lingua franca, multilingualism has come to predominate. The large number of trilinguals and multilinguals in the world has led to a multilingual turn (Ortega, 2013), and third language acquisition research has been recognized as an independent and essential area in applied linguistics in recent years (e.g, Huang et al., 2020; Jessner et al., 2021).

Lexical richness, a multidimensional construct that consists of lexical sophistication, lexical diversity, and lexical density, has been recognized as one of the most effective methods for assessing learners' writing proficiency and development (e.g, Laufer and Nation, 1995; Read, 2000; Zhang, 2020; Zhang et al., 2021). A well-written text is often the result of a writer's careful selection and proper use of words (Zhang et al., 2021). Specifically, lexical sophistication refers to the proportion of infrequent or advanced words in a text; lexical diversity refers to the variety of words used in the text; and lexical density refers to the proportion of lexical words in a text (Read, 2000). Therefore, it is also assumed that advanced learners will use more sophisticated, diverse, and appropriate words in their writings (Zhang et al., 2021). Consequently, lexical richness is an important aspect in writing-related research.

Existing studies of lexical richness in writing research may be classified into three categories. Studies in the first category attempted to investigate learners' lexical richness levels compared with those of native English speakers (e.g., Fairclough and Belpoliti, 2016; Eckstein and Ferris, 2018; Lei and Yang, 2020). Research in the second category examines the relationship between lexical richness and writing quality (e.g., Zhu, 2013; Kim et al., 2018; Lee et al., 2021). However, these studies yielded inconsistent results with respect to the relationship between the measures of lexical richness and writing quality.

The third category of studies has sought to explore lexical richness in written texts from the developmental perspective. Numerous studies in this vein have investigated one or two measures along with other linguistic features (e.g., Higginbotham and Reid, 2019; Bulté and Housen, 2020; Wang and Wang, 2020; Zhang and Daller, 2020). For instance, lexical diversity and syntactic complexity have been examined in writing samples by secondary school learners (Bulté and Housen, 2020). Lexical sophistication and lexical diversity have been measured in argumentative texts by college-level learners (Wang and Wang, 2020). Lexical sophistication has been exclusively investigated in academic writing by advanced L2 learners (Higginbotham and Reid, 2019). Additionally, several studies have explored the developmental features of lexical richness holistically, while most have focused primarily on L2 learners and yielded rather conflicting results. Researchers have reported cross-sectional studies that identify the developmental features of lexical richness (e.g., Deng, 2020; Zhang, 2020; Zhang et al., 2021). For instance, Deng (2020) compared lexical differences in L2 English writing at different proficiency levels. The results revealed that lexical sophistication and lexical diversity increased with proficiency level, while lexical density showed a non-linear developmental trend. Zhang et al. (2021) compared lexical differences in L2 English writing by Chinese beginner learners across three grade levels. The results demonstrated that all four lexical richness measures increased with grade levels. However, these studies yielded inconsistent results regarding the developmental features of lexical richness. Furthermore, numerous studies have explored how lexical richness develops over time in L2 writing (Wang and Zhou, 2012; Zhu and Wang, 2013; Zheng, 2015, 2016). For example, Zheng (2016) investigated the development of lexical richness in L2 writing by university students over the course of 1 year. The results revealed that while lexical density plateaued, lexical sophistication and lexical diversity increased over time.

Despite growing interest in the developmental features of lexical richness, research participants are limited to L2 learners at different proficiency levels (e.g., Higginbotham and Reid, 2019; Wang and Wang, 2020; Zhang et al., 2021). Much remains to be known about how lexical richness develops over time in L3 learners' writing, particularly for L3 beginner learners. Given the role of learning contexts in language learning, the developmental features of lexical richness in L3 writing may differ from those in L2 writing. Moreover, beginner learners are more likely than advanced learners to experience difficulties with lexical choices (Fairclough and Belpoliti, 2016; Zhang et al., 2021). Finally, according to the dynamic usage-based (DUB) approach, variation is ever present among learners, even those at the same stage of development (Verspoor et al., 2012). Therefore, it is necessary to investigate variations in lexical richness in L3 beginner learners' written samples and the dynamic process of L3 writing development.

For these reasons, it is of particular importance to investigate the developmental features of lexical richness in L3 beginner learners' writings. The present study performs cross-sectional comparisons of the developmental features of the three dimensions of lexical richness (namely, lexical sophistication, lexical diversity and lexical density) in English writings by Chinese L3 beginner learners across three learning stages from the perspective of the DUB approach. The results have implications for L3 vocabulary teaching in secondary schools. Specifically, we addressed the following three research questions.

RQ1: What are the developmental features of lexical sophistication in English writing by Chinese L3 beginner learners?
RQ2: What are the developmental features of lexical diversity in English writing by Chinese L3 beginner learners?
RQ3: What are the developmental features of lexical density in English writing by Chinese L3 beginner learners?

To the best of our knowledge, our study is the first to compare the developmental features of lexical richness in L3 writing by Chinese L3 beginner learners in secondary schools. The findings from this study are expected to contribute to research on L3 writing development by providing the developmental features of lexical richness in L3 writings by L3 beginner learners of English. In the next section, we provide the theoretical framework. Subsequently, we detail the materials and methods used. The results and discussion will be presented in the following two sections. Finally, we offer several conclusions.

## Theoretical Framework

The DUB approach generated by Langacker (2000) is a theory which combines the usage-based approach and dynamic systems theory. It operates on the principle that the language system is characterized by non-linearity, dynamics and interconnectedness (Larsen-Freeman and Cameron, 2008). Language development is influenced not only by internal factors, such as motivation, aptitude, and learner's age, but also external factors, such as language input, language experience, learning context, and the time invested in language learning. In line with this concept, Ortega (2014) generated the language exposure hypothesis, which holds that language exposure plays a decisive role in language learning. Specifically, the more language exposure the learners receive, the higher their language proficiency will ultimately become (Verspoor and Smiskova, 2012).

The DUB approach also aligns with dynamic systems theory. It assumes that language systems consist of many interconnected subsystems, which indicates a change in one subsystem will also promote the development of the whole language system. Consequently, language systems constantly undergo dynamic changes during the language development process, which is characterized by variation and variability. These are considered to be key motors of language development and change (Bulté and Housen, 2018). The former refers to language development across group levels, while the latter refers to language development within individual learners. As DUB approach claims, variation always emerges among learners, even those at the same developmental stage (Verspoor et al., 2012).

Adopting the DUB approach, we assessed L3 beginner learners' writing samples to obtain insight into the variation of lexical richness and the dynamic process of L3 writing development using cross-sectional data, with the written samples representing different learning stages of the developmental process in L3 writing.

## Materials and Methods

### Corpus Description

The writing samples used in the present study were collected from the Writing Corpus of Chinese Ethnic Minority Beginner Learners as the Third Language (WCCMBL), which was compiled by the School of Foreign Languages at Northeast Normal University in the spring semester of 2020. The corpus contains English compositions written by Chinese ethnic minority L3 beginner learners of English aged 13–18. The compositions in the corpus are descriptive. The writing samples are on the same topics, with which the participants are familiar: self-introduction, introduction to career planning and introduction to traditional festivals. During a 30-min period in class, participants were assigned these topics simultaneously and chose one as the subject of their written assignment, which ranged from 50 to 100 words and completed without the assistance of any reference materials. Altogether, the corpus comprises 1,201 writing samples with 121,237 tokens.

To attain the purpose of this study, 619 written texts by secondary school students across three learning stages (stage 1: grades 7–8; stage 2: grades 9–10; stage 3: grades 11–12) were sampled from the WCCMBL. The sample sizes of three learning stages were almost the same. The stage 1, stage 2, and stage 3 samples contained 19,955, 20,530, and 20,022 tokens, respectively.

### Participants

The writing samples used in this study were produced by Korean minority students in junior and senior high schools. These students all grew up in Yanbian Korean Autonomous Prefecture, Jilin Province, and their L1 is Korean. All participants have the same language learning background: specifically, they learn Korean (L1) and Chinese (L2) simultaneously in grade 1, which is the first year in primary school, and begin to learn English as a third language in grade 3. As the participants usually communicate with their parents in two languages, their exposure to English outside the classroom is extremely limited. Consequently, Chinese students learn English mainly through classroom instruction (Zhang et al., 2021). The participants had been receiving formal instruction for an average of 3 years prior to entering secondary school. During this period, students only learned the alphabet and were able to make daily conversations (Jiang et al., 2019). However, since students' writing scores are a key method for assessing English ability, they receive formal English writing instruction from grade 7, which is the first year of secondary school. As a key aspect of English teaching, English writing instruction is offered during one or two periods every 2 weeks (Zhang et al., 2021). Therefore, the participants in the present study—junior high school students and senior high school students—may be regarded as L3 beginner learners of English writing. The participants were all informed that their written samples would only be used for academic purposes, and they were willing to be part of the corpus construction project.

### Lexical Richness Measures

A vast body of research into the operationalization of lexical richness exists in the literature (Laufer and Nation, 1995; Read, 2000; Daller et al., 2003; Jarvis, 2013). Several scholars have defined lexical richness as lexical complexity or lexical diversity (Daller et al., 2003; Bulté and Housen, 2012). However, Laufer and Nation (1995) suggest that lexical richness comprises four dimensions: lexical sophistication, lexical originality, lexical density, and lexical variation. Read (2000) considers that lexical richness includes lexical sophistication, lexical density, lexical variation, and lexical errors. Bulté and Housen (2012) also define four dimensions of lexical complexity: lexical density, lexical diversity, lexical sophistication, and lexical compositionality. As indicated above, lexical richness typically comprises three dimensions: lexical sophistication, lexical density and lexical diversity. Therefore, the present study traces the developmental features of these three measures in Chinese L3 beginner learners' English writing samples.

### Lexical Sophistication

A text's lexical sophistication is usually measured by the proportion of infrequent or advanced words it contains (Read, 2000). High-frequency words are generally considered to be basic, while low-frequency words are considered to be more advanced (e.g., Laufer and Nation, 1995; Zheng, 2016; Zhang et al., 2021). Lexical sophistication is also related to the degree of formality of writing (Qin and Wen, 2007; Zhang et al., 2021). That is, the more basic and frequent the words are, the more informal and spoken-like the writing will be (Zhang et al., 2021).

Lexical Frequency Profile (LFP) (Laufer and Nation, 1995) is one of the most widely used measures in terms of words' frequency, which divides the texts into four frequency bands: the first 1,000 most frequent words, the second 1,000 most frequent words, the academic word list (AWL), and not-in-the-lists words (Coxhead, 2000). Learners' correct use of words in the AWL and not in the lists has been defined as low-frequency words (Laufer and Nation, 1995). Accordingly, the proportion of low-frequency words used in the text is an indicator of lexical sophistication (Zhang et al., 2021). However, the words in LFP exceed beginner learners' proficiency (Laufer and Nation, 1995), and advanced words defined by AWL rarely appear in beginner learners' written samples (Zhang et al., 2021). Consequently, it is crucial to develop an appropriate word list to measure lexical sophistication (Liang et al., 2010). Zhang et al. (2021) have developed a new base list for secondary students based on the words in the English textbooks published by the Chinese People's Education Press. In their study, the words in the textbooks were classified into three frequency bands: the word lists in textbooks for grades 7–9 correspond to base list 1, base list 2, and base list 3. In the present study, we will adopt similar base lists to examine the developmental features of lexical sophistication. The base lists were selected for two reasons: first, learners who are taught in classroom settings fundamentally learn words from these textbooks (Ellis, 2013). Second, under the guidance of the Ministry of Education of the People's Republic of China, the textbooks for L2 and L3 beginner learners are identical. As the new base list for secondary students can effectively measure L2 beginner learners' lexical sophistication (Zhang et al., 2021), it is also applicable for investigating L3 beginners' lexical sophistication levels in the present study.

### Lexical Diversity

Lexical diversity, also called lexical variation (e.g., Read, 2000; Zhang et al., 2021), refers to “the variety of the different words used in a text” (Read, 2000. p. 200). Learners with various lexical repertoires are more likely to choose different words in their writing (Zhang et al., 2021). Hence, learners' productive word repertoires can be measured effectively in terms of lexical diversity (Read, 2000).

Different indices have been developed to measure lexical diversity, such as type-token ratio (TTR), Uber index and D. TTR is an index that is often used when the length of text remains constant (Zhang et al., 2021). In addition, TTR can be calculated more easily than other indices. Therefore, this study maintained the sample sizes of three learning stages almost the same and used TTR to measure the lexical diversity of L3 beginner learners' writings.

### Lexical Density

Lexical density is typically defined as “the proportion of lexical words found in a text” (Read, 2000, p. 203), reflecting the information content of a text (Biber et al., 2012). Therefore, an abundance of lexical words in a text signifies its high information load (Zhang et al., 2021). In addition, lexical density is a good way to describe the level of a text along the oral-written continuum (Halliday, 2002, p. 329). It is generally true that a text with a higher lexical density is typically more formal. Lexical density increases as the text moves along the continuum from spoken to written (Zhang et al., 2021).

### Data Analysis

As indicated above, variation always emerges among learners even at the same stage of development (Verspoor et al., 2012). Therefore, by following traditional cross-sectional methods (e.g., Jiang et al., 2019; Zhang et al., 2021), we examined the developmental features of lexical richness in L3 written texts based on writing samples across three learning stages to identify variation in L3 writing development. The log-likelihood (LL) test was performed to investigate the significant differences across these three learning stages. Significant levels were defined as follows: sig. (*p*) = significance (*p*); *p* < 0.05, critical value = 3.84; *p* < 0.01, critical value = 6.63; *p* < 0.001, critical value = 10.83; *p* < 0.0001, critical value = 15.13 (Liang et al., 2010; Zhang et al., 2021).

### Developmental Features of Lexical Sophistication

First, in accordance with Zhang et al.'s study (2021), we developed new base lists for secondary school students. As the vocabulary sequences in the textbooks follow the natural order of lexical learning—that is, from frequent words to infrequent words (Zhang et al., 2021)—this study classified the vocabulary in the textbooks into three frequency bands. Specifically, word lists in the textbooks for learning stages 1–3 corresponded to base lists 1, 2, and 3.

Second, the present study adopted the Range program to estimate the distribution of words from different word lists in L3 writings. To prevent the Range program from classifying the proper nouns, words, and misspellings in the written samples as the not-in-the-lists words, proper nouns and words were manually tuned to high-frequency words and misspellings were corrected in the text. After submitting L3 beginner learners' writing samples to the Range program, the software generated the L3 Beginner Learners' Vocabulary List by comparing the samples with three base lists. The vocabulary list composed of four sublists, namely, word list 1, word list 2, word list 3 and not in the lists. In line with LFP, the percentage of the words in the word list 1 and word list 2 are considered to be high-frequency words, whereas the percentage of the words in the word list 3 and not in the lists are taken as low-frequency words.

Third, we calculated the number of words types across three learning stages and then calculated the number and percentages of low-frequency word types. Finally, LL tests were conducted to determine whether significant differences were evident across the three learning stages.

### Developmental Features of Lexical Diversity

This study utilized TTR to investigate lexical diversity using AntConc 3.5.7 (Anthony, 2004). First, TTR was calculated for each learning stage. Second, LL tests were used to compare the differences of lexical diversity across the three learning stages to find the developmental features of lexical diversity.

### Developmental Features of Lexical Density

This study adopted the ratio of lexical words in a text to measure the degree of lexical density. Lexical words consisted of nouns, verbs, adjectives, and adverbs (Zhang et al., 2021). First, the written samples were tagged using Treetagger. We then used AntConc 3.5.7 to extract each class of lexical words, count the number of tokens within each class, and determine the total number of lexical words. Second, LL tests were used to examine whether differences in the ratio and proportion of lexical word classes were significant across the three learning stages.

## Results

Based on cross-sectional comparisons among writing samples across three learning stages, this section presents the developmental features of lexical sophistication, lexical diversity, and lexical density identified in L3 beginner learners' English writings using the DUB approach.

### Lexical Sophistication

Our first research objective was to investigate the developmental features of lexical sophistication in L3 writings by Chinese L3 beginner learners of English. For this question, the overall level of lexical sophistication in the writing samples of three learning stages was examined first. The developmental trend of lexical sophistication across the three learning stages was then investigated. Table 1 presents the results.

**Table 1:** Percentage of word types per learning stage.

**Learning stages**	**Word list 1 (%)**	**Word list 2 (%)**	**Word list 3 (%)**	**Not in the lists (%)**
Stage 1	82.44	2.94	0.53	14.09
Stage 2	74.96	10.03	1.34	13.67
Stage 3	72.22	11.46	3.63	12.70

As Table 1 illustrates, high-frequency word types account for 85.38, 84.99, and 83.68% of the total number of types in the written samples from the three learning stages, whereas low-frequency word types account for 14.62, 15.01, and 16.32%. Based on the comparison of the two sets of data, the participants are more likely to use high-frequency words in L3 writing, and their productive control of low-frequency words remains limited.

Additionally, as the high-frequency word ratio denotes the degree of informality in writing (Qin and Wen, 2007; Zhang et al., 2021), the high ratios of frequent words observed in this study demonstrate that L3 beginner learners' writings are informal in style and spoken-like in register. It can be further supported by the written samples. As Example 1 illustrates, the extensive use of pronouns, modal verbs and imperatives in Chinese L3 beginner learners' writing samples is indicative of the informality of their writing (Zhu, 2013; Zhang et al., 2021). Formality in writing appears to be one of the greatest challenges that L3 beginner learners encounter.

1. Career planning is important for students. First of all, *my* dream is become a teacher. *I* think educate is very important. *They can* fell fun. *I can* get along with students and *my* learn ability is good. *I* will study hard for *my* dream. *I* will be students lead to study. (WCCMBL-2-0105.txt)

Regarding the developmental trend of lexical sophistication, the low-frequency word ratio in the written samples indicates positive growth, with a ratio of 14.62% for stage 1, 15.01% for stage 2, and 16.32% for stage 3 (see Table 1). The gradual increase in lexical sophistication is consistent with earlier studies' findings (Laufer and Nation, 1995; Fairclough and Belpoliti, 2016; Zhang, 2020; Zhang et al., 2021).

However, as Table 2 illustrates, LL tests reveal that no significant increases in lexical sophistication between adjacent or non-adjacent learning stages (stages 1 and 2: LL = −0.06, *p* = 0.813; stages 2 and 3: LL = −0.68, *p* = 0.408; stages 1 and 3: LL = −1.00, *p* = 0.317). Rather, the results indicate slow progress in lexical sophistication as illustrated by L3 beginner learners' written samples. Close examination of the two low-frequency word lists reveals that the increases in word list 3 are significant between adjacent or non-adjacent learning stages (stages 1 and 2: LL = −3.93, *p* = 0.047; stages 2 and 3: LL = −13.56, *p* = 0.000; stages 1 and 3: LL = −26.87, *p* = 0.000). By comparison, the increases in not in the lists are non-significant between adjacent or non-adjacent learning stages (stages 1 and 2: LL = −0.07, *p* = 0.790; stages 2 and 3: LL = 0.44, *p* = 0.506; stages 1 and 3: LL = 0.78, *p* = 0.378). The gradual increase in lexical sophistication appears to result from the word types present in word list 3.

**Table 2:** Number of word types per word list and per learning stage.

**Learning stages**	**Word list 3**		**Not in the lists**		**Total (lexical sophistication)**	
	**LL**	***p***	**LL**	***p***	**LL**	***p***
Stages 1–2	−3.93	0.047	−0.07	0.790	−0.06	0.813
Stages 2–3	−13.56	0.000	0.44	0.506	−0.68	0.408
Stages 1–3	−26.87	0.000	0.78	0.378	−1.00	0.317

*LL, log-likelihood test; sig. (p), significance (p)*.

To summarize, an abundance of high-frequency words used by L3 beginner learners in their writing indicates the informality and spoken style of their writing. However, L3 beginner learners make gradual progress in lexical sophistication by increasing their use of low-frequency words, despite non-significant improvements across the three learning stages.

### Lexical Diversity

Our second research question addressed the developmental features of lexical diversity in L3 writings by Chinese L3 beginner learners of English. To address this question, we first investigated the overall level of lexical diversity in writing samples from the three learning stages. Next, the developmental trend of lexical diversity across three learning stages was explored. The results are presented in Table 3.

**Table 3:** Type-token ratio (TTR) per learning stage.

**Learning stages**	**Types**	**Tokens**	**TTR**	**LL**
Stage 1	926	19,955	4.64%	Stages 1 and 2: LL = −31.80, *p* = 0.000
Stage 2	1,217	20,530	5.93%	Stages 2 and 3: LL = 0.02, *p* = 0.887
Stage 3	1,180	20,022	5.89%	

*LL, log-likelihood test; sig. (p), significance (p)*.

Table 3 presents the TTR results for the written samples across the three learning stages as well as LL texts between adjacent learning stages. As the table illustrates, the TTR is relatively low for all three learning stages, at 4.64, 5.93, and 5.89%, respectively. Based on these low values, it is clear that the written samples produced by L3 beginner learners are not lexically diverse. L3 beginner learners are prone to use similar lexical items repeatedly in their writing. This result is line with those of Fairclough and Belpoliti (2016) and Zhang et al. (2021), who also reported low levels of lexical diversity in L2 beginner learners' written samples.

Regarding developmental trends in lexical diversity, Table 3 indicates that TTR develops non-linearly as learning stages increase (stages 1 and 2: 4.64% < 5.93%; stages 2 and 3: 5.93% > 5.89%). LL tests demonstrate that the increase is significant from stage 1 to 2 (LL = −31.80, *p* = 0.000), whereas the decrease is non-significant from stage 2 to 3 (LL = 0.02, *p* = 0.887). These results reveal that L3 beginner learners across three learning stages vary their lexical choices in their writings.

Furthermore, the number of word types per word list was also compared between adjacent learning stages. As can be seen in Table 4, with regard to stage 1 and 2, the number of word types in word lists 1–3 significantly increase over time. Specifically, the number of word types rise from 784 to 949 for word list 1 (LL = −10.82, *p* = 0.001), from 28 to 127 for word list 2 (LL = −65.26, *p* = 0.000), and from 5 to 17 for word list 3 (LL = −6.53, *p* = 0.011). That is, the TTR of word lists 1–3 significantly increase from stage 1 to 2. By comparison, the number of word types slightly increase from 134 to 173 for not in the lists (LL = −3.78, *p* = 0.052). In terms of stage 2 and 3, the number of words from list 1 and not in the lists decline non-significantly. Specifically, the number of word types decline from 949 to 876 for word list 1 (LL = 1.29, *p* = 0.256), and from 173 to 154 for not in the lists (LL = 0.65, *p* = 0.419). By comparison, the number of word types in the other two lists increase slightly and significantly. Specifically, the number of word types increase from 127 to 139 for word list 2 (LL = −0.91, *p* = 0.340), and from 17 to 44 for word list 3 (LL = −13.11, *p* = 0.000). Taken together, these results indicate that participants in the three learning stages exhibit significant differences in their lexical choices. That is, learners in lower learning stages (stages 1 and 2) tend to use basic vocabulary from word list 1, whereas learners in learning stage 3 tend to use more diverse vocabulary from beyond word list 1 when writing English compositions. Consequently, the compositions become lexically sophisticated and varied over time, which is also consistent with the increase in lexical sophistication.

**Table 4:** Number of word types per word list and per learning stage.

**Learning stages**	**Word list 1**	**Word list 2**	**Word list 3**	**Not in the lists**
Stage 1	784	28	5	134
LL	LL = −10.82, *p* = 0.001	LL = −65.26, *p* = 0.000	LL = −6.53, *p* = 0.011	LL = −3.78, *p* = 0.052
Stage 2	949	127	17	173
LL	LL = 1.29, *p* = 0.256	LL = −0.91, *p* = 0.340	LL = −13.11, *p* = 0.000	LL = 0.65, *p* = 0.419
Stage 3	876	139	44	154

*LL, log-likelihood test; sig. (p), significance (p)*.

To conclude, L3 beginner learners showed a lack of varied lexical selection. However, as the learning stages increased, lexical diversity also initially increased significantly, and then declined slightly. A lexical plateau then seemed to occur. In addition, distinctive developmental features are evident in written English compositions: L3 learners in lower learning stages tend to use diverse basic words from word list 1, whereas those in learning stage 3 incorporate more diverse and sophisticated words from beyond word list 1.

### Lexical Density

Our third research question explored the developmental features of lexical density in L3 writing by Chinese L3 beginner learners of English. To answer this question, we first examined the overall level of lexical density in the writing samples across three learning stages. We then investigated the developmental trend of lexical density across the three learning stages.

As Table 5 illustrates, lexical density ratio of 41.58, 44.34, and 46.86% was observed in the written samples across the three learning stages. These ratios are lower than those observed in similar studies that focused on L2 beginner, intermediate, and advanced learners. For instance, some researchers have found that the ratio of lexical density is close to 60% in Chinese English major students' writing (Zhu and Wang, 2013; Zheng, 2016). However, the lexical density of L3 beginner learners is relatively low, indicating the general simplicity of their writing's content. For instance, Zhang et al. (2021) also found that Chinese junior high school L2 beginner learners of English manifested percentages of 41.37, 43.71, and 43.93% in their written samples.

**Table 5:** Lexical density per learning stage.

**Learning stages**	**Lexical words tokens**	**Tokens**	**Lexical density**	**LL**
Stage 1	8,298	19,955	41.58%	stages 1 and 2: LL = −17.96, *p* = 0.000
Stage 2	9,104	20,530	44.34%	Stages 2 and 3: LL = −14.10, *p* = 0.000
Stage 3	9,383	20,022	46.86%	

*LL, log-likelihood test; sig. (p), significance (p)*.

Regarding the developmental trend of L3 beginner learners' lexical density, Table 5 demonstrates that the ratio of lexical words according to learning stages (41.58% < 44.34% < 46.86%). LL tests demonstrate significant increases between adjacent learning stages (stages 1 and 2: LL = −17.96, *p* = 0.000; stages 2 and 3: LL = −14.10, *p* = 0.000). These results indicate that English compositions by L3 beginner learners exhibit higher content over time, moving from the informal, spoken-like register to a formal, written-like register across the three learning stages. This positive growth in lexical density mirrors the findings of earlier studies that focused on L2 beginner, intermediate, and advanced learners (Bao, 2008; Zheng, 2016; Zhang, 2020; Zhang et al., 2021).

The distribution of lexical word classes was further analyzed across the three learning stages. As Table 6 illustrates, lexical word classes are unevenly distributed throughout the written samples of L3 beginner learners. The most frequent lexical word class used by L3 beginner learners is nouns (39.04%), followed sequentially by verbs (36.45%), adjectives (13.47%), and adverbs (11.03%). The results indicate that L3 beginner learners tend to use more nouns in the lower learning stages. This overreliance on some lexical classes, such as nouns, suggests that L3 beginner learners experience difficulties in selecting from the diverse lexical word classes in a given context. However, it may also indicate insufficient in-class instruction of lexical word classes. These results support earlier studies' findings on L2 beginner learners' propensity for using nouns over verbs (Yoshida, 1978; Zhang, 2020; Zhang et al., 2021).

**Table 6:** Distribution of lexical word classes across learning stages.

**Learning stage**	**Nouns**		**Verbs**		**Adjectives**		**Adverbs**	
	**Frequency/percentage**	**Normalized frequency**	**Frequency/percentage**	**Normalized frequency**	**Frequency/percentage**	**Normalized frequency**	**Frequency/percentage**	**Normalized frequency**
1	3,733/44.99%	187.07	2,957/35.64%	148.18	951/11.46%	47.66	657/7.92%	32.94
2	3,463/38.04%	168.68	3,093/33.97%	150.66	1,421/15.61%	69.22	1,127/12.38%	54.90
3	3,261/34.75%	162.87	3,713/39.57%	185.45	1,237/13.17%	61.78	1,172/12.49%	58.54
Total	39.04%		36.45%		13.47%		11.03%	

In addition, the distribution of lexical word classes varies across the three learning stages (see Tables 6, 7). The normalized frequency of nouns decreases from learning stage 1 to 3 (187.07 > 168.68 > 162.87). LL tests indicate that these declines are significant between learning stages 1 and 2 (LL = 19.25, *p* = 0.000), but non-significant between learning stages 2 and 3 (LL = 2.06, *p* = 0.151). Unlike the downward trend of nouns, verbs and adverbs show upward trends over time (verbs: 148.18 < 150.66 < 185.45; adverbs: 32.94 < 54.90 < 58.54). LL tests demonstrate that the increase in verbs is non-significant from learning stage 1 to 2 (LL = −0.41, *p* = 0.520) but significant from learning stage 2 to 3 (LL = −73.16, *p* = 0.000). Regarding the adverbs, the increases are significant from learning stage 1 to 2 (LL = −112.30, *p* = 0.000), but non-significant from learning stage 2 to 3 (LL = −2.37, *p* = 0.124). Regarding the developmental trend of adjectives, they show non-linear upward trends over time (47.66 < 69.22 > 61.78). LL tests demonstrate that the increases in adjectives are significant between adjacent learning stages (stages 1 and 2: LL = −80.87, *p* = 0.000; stages 2 and 3: LL = 8.55, *p* = 0.003). Overall, L3 beginner learners in the upper learning stages exhibit less reliance on nouns, and tend to use other lexical word classes (verbs, adjectives, adverbs) in their writing composition. In other words, lexical selection and use of lexical word classes is shown to be improved in L3 beginner learners' writing samples over time.

**Table 7:** Log-likelihood (LL) tests for lexical word classes across learning stages.

**Learning stages**	**Nouns**		**Verbs**		**Adjectives**		**Adverbs**	
	**LL**	***p***	**LL**	***p***	**LL**	***p***	**LL**	***p***
Stages 1–2	19.25	0.000	−0.41	0.520	−80.87	0.000	−112.30	0.000
Stages 2–3	2.06	0.151	−73.16	0.000	8.55	0.003	−2.37	0.124
Stages 1–3	33.48	0.000	−83.36	0.000	−36.54	0.000	−145.27	0.000

*LL, log-likelihood test; sig. (p), significance (p)*.

In summary, L3 beginner learners use relatively fewer lexical words. However, a significantly positive growth of lexical density is observed across the three learning stages. In addition, L3 beginner learners use more diverse lexical word classes rather than relying excessively on nouns across three learning stages.

## Discussion

**Figure 1:**
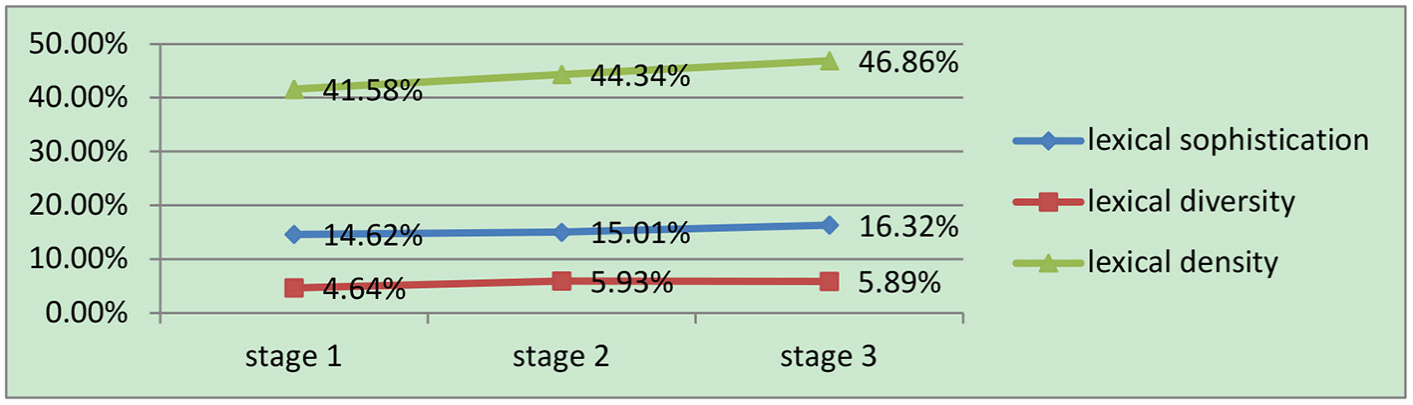
The development of lexical richness in L3 writing.

The present study is the first to investigate the developmental features of lexical richness in written samples by Chinese L3 beginner learners of English in secondary schools across the three learning stages. Based on the developmental features of lexical sophistication, lexical diversity, and lexical density analyzed above, the results indicate a dynamic development of lexical richness in L3 writings, with each of the three measures developing unevenly, as illustrated by Figure 1. Therefore, the possible reasons for these developmental features of lexical richness from the perspective of the DUB approach are addressed in the section that follows.

### Lexical Sophistication

As observed above, Chinese L3 beginner learners are likely to use numerous high-frequency words in their writing and their general level of lexical sophistication remains at beginner's level. This supports the finding of overreliance on high-frequency words in learners' writing (Laufer and Nation, 1995; Fairclough and Belpoliti, 2016; Zhang et al., 2021). Laufer and Nation (1995) indicate that beginner learners' writing is characterized by an overwhelming percentage of high-frequency words. Zhang et al. (2021) additionally found that Chinese L2 beginner learners used a high percentage of high-frequency words in their writings.

Furthermore, research has shown that more formal lexical words are frequently used in native-like writing (Schmitt, 2000). Chinese L3 beginner learners prefer to use high numbers of high-frequency words, which suggests a low level of formality in L3 writing. As previous studies have shown, even advanced Chinese learners also use numerous high-frequency words in their writings (Qin and Wen, 2007; Zheng, 2016; Wang and Wang, 2020). Therefore, the use of formal or low-frequency words for L3 beginner learners probably develops slowly. According to the DUB approach, language acquisition will be influenced by the degree of language input. As the low-frequency words progress slowly, greater language exposure is required to acquire them. Therefore, for learners to improve the formality of their writing, they require abundant exposure to low-frequency words.

As analyzed above, the lexical sophistication of Chinese L3 beginner learners increases across the three learning stages, albeit generally at a significantly lower level. Nevertheless, the lexical sophistication of Chinese L3 beginner learners exhibits unique developmental patterns in comparison with earlier studies (e.g., Laufer and Nation, 1995; Zhu and Wang, 2013; Wang and Wang, 2020; Zhang et al., 2021). That is, the lexical sophistication of Chinese L3 beginner learners progresses slowly every year between adjacent learning stages: that is, the lower learning stages (stages 1–2) and upper learning stages (stages 2–3) both develop slowly. However, earlier studies have found that learners made significant progress in lexical sophistication over time. The discrepancy may be explained by the different vocabulary profiles used in the present study. Most earlier studies investigated the developmental features of lexical sophistication with LFP. In contrast to these studies, this study uses the Beginners' Writing Vocabulary List, which has been shown to be more appropriate for Chinese beginner learners (Zhang et al., 2021). As noted earlier, the Beginners' Writing Vocabulary List has been adopted from Zhang et al.'s (2021) study, but the developmental trends of lexical sophistication observed in the present study are quite different from their study. In their study, a significant growth trend was observed in lexical sophistication by Chinese L2 beginner learners across three grade levels (grades 7–9). One possible explanation for this discrepancy is that the different groups of learners were studied. Unlike their study, this study investigates L3 beginner learners. As Herdina and Jessner (2002) indicated, third language acquisition is more complicated than second language acquisition owing to the interaction between internal and external factors, such as individual factors, learning contexts, and social factors, which is also in line with the DUB approach. One is that language attrition is one of the individual factors cannot be ignored in L3 learning. Multilingual learners have been shown to exhibit more frequent language attrition than bilingual learners (Jessner, 2006; Jessner et al., 2021). As L3 beginner learners have lower proficiency levels, they may experience more language attrition in L3 learning. Despite the greater linguistic exposure over time, lexical sophistication develops slowly across the three learning stages. Furthermore, the discrepancy is likely to contribute to dynamic competition between the three languages. In the present study, L3 beginner learners should not only develop L1 and L2 lexical sophistication but should also make progress in L3 lexical sophistication. Additionally, owing to the complexity of language systems, the time invested in L3 lexical learning is also limited. Therefore, in the course of learning low-frequency words, L3 beginner learners are likely to confront more difficulties, so they should make more effort to maintain L3 lexical development. According to the DUB approach, as language input is one of the fundamental factors for language development, extensive linguistic exposure is required for L3 beginner learners to make substantial improvements in using low-frequency words.

### Lexical Diversity

As noted above, lexical diversity in L3 beginner learners' writing is generally low. One explanation for this lower lexical diversity may be related to the limited linguistic exposure to words and nature of lexical learning (Zhang et al., 2021). As L3 beginner learners are still at the initial stages of L3 writing, their exposure to varied words is significantly less sufficient. Furthermore, as lexical learning is receptive in nature (Tracy-Ventura, 2017), L3 beginner learners who are in the initial stages of lexical learning are likely to learn vocabulary receptively. Consequently, they may encounter difficulties in choosing the appropriate words to express their ideas. Therefore, lexical diversity in L3 beginner learners' writing remains at a lower level.

However, lexical diversity in L3 beginner learners' writing increases unevenly across the three learning stages. That is, it increases significantly from stage 1 to 2 but slowly decreases from stage 2 to 3. Nevertheless, lexical diversity in learning stage 3 is higher than that in learning stage 2. This suggests that lexical diversity in L3 beginner learners' writing develops non-linearly over time, which is also consistent with the concept of the DUB approach. As Ellis (2013) observed, learners will learn more words with increased language exposure, but their productive vocabulary develops slowly. Therefore, it is extremely difficult for L3 beginner learners to produce more diverse words in their writings. It aligns with earlier studies that focused on advanced learners (e.g., Zheng, 2016; Wang and Wang, 2020). However, the rate of increase between adjacent groups varies (Zhang, 2020; Zhang et al., 2021). For instance, Zhang et al. (2021) found that lexical diversity of Chinese L2 beginner learners increased significantly every school year. The different group of learners studied is one possible explanation for discrepancy in findings. Unlike their study, which focused on L2 beginner learners, the present study investigated L3 beginner learners. As noted above, owing to the complexity of language systems, L3 beginner learners encounter greater difficulties in learning L3 vocabulary. Therefore, the level of lexical diversity is lower than that of L2 beginner learners. However, with increased exposure to diverse words, L3 beginner learners in lower learning stages develop significantly. In addition, L3 beginner learners in upper learning stages are likely to have reached a lexical plateau in the process of lexical learning (Zhang et al., 2021). Thus, the lexical diversity of L3 beginner learners decreases slowly from stage 2 to 3. Therefore, extra exposure to varied words is required to improve lexical diversity during learning stage 3.

In addition, the diverse frequent words used by L3 beginner learners in lower learning stages were traceable to base list 1, whereas various infrequent words employed by learning stage 3 were selected from beyond word list 1. According to the DUB approach, increased frequency of linguistic exposure in diversified contexts can promote efficiency in language learning. In Chinese L3 classrooms, most teachers implement multiple types of lexical learning activity, such as reading and vocabulary exercises, to ensure sufficient exposure to a widely varying vocabulary. Hence, L3 beginner learners can learn and produce more various words gradually over time.

Although the lexical diversity of L3 beginner learners has increased gradually, different learning stages show diversified developmental trends. Specifically, learning stages 1 and 2 have been learning the words in base list 1 and 2, so they are more prone to use words from these lists. However, learners in stage 3 have studied and come across more complicated words, so they can produce more diverse and complex words beyond words from word list 1 and 2.

### Lexical Density

As observed above, Chinese L3 beginner learners have generally low lexical density. This is in line with the cognitive rule of language acquisition. As mentioned in an earlier section, lexical density can be used to distinguish levels of texts in the oral-written continuum (Halliday, 2002). That is, the more lexical words that are used in the texts, the more formal the text is likely to be. Accordingly, the relatively lower level of lexical density in L3 beginner learners' writing indicate a less formal and spoken-like register, which suggests that they use formal words less frequently in their writing (Schmitt, 2000). According to the DUB approach, insufficient language input will decelerate language acquisition. Owing to the reduced linguistic exposure to lexical words in the classroom, the lexical density of L3 beginner learners is relatively low. Additionally, because of the competition of language systems and the limitations of available resources (Herdina and Jessner, 2002), L3 beginner learners experience difficulties with appropriate lexical word use. Therefore, extra exposure to lexical words is a necessity to improve lexical density in L3 writing.

As analyzed previously, despite the fact that L3 beginner learners have generally low lexical density, it increases significantly across the three learning stages. The positive growth in lexical density is consistent with previous results (Zheng, 2016; Wang and Wang, 2020; Zhang, 2020; Zhang et al., 2021). For instance, Zhang et al. (2021) found that lexical density of Chinese L2 beginner learners increased every school year. Although language learners in their study are different from this study, the lower level of lexical density in beginner learners' writings is similar, which is congruent with general cognitive rules of language acquisition. Beginner learners have limited abilities to use lexical words to produce the texts with higher information content.

However, the growth rate of lexical density differs across the group levels. For instance, Wang and Wang (2020) found that lexical density developed non-linearly, whereas Zhu and Wang (2013) reported general progress every school year with no significant differences between the adjacent groups. According to the DUB approach, language development is influenced by both internal factors and external factors. The discrepancy may be explained by the different learners' language proficiency levels. Unlike earlier studies, which focused on intermediate and advanced learners, the present study investigated L3 beginner learners. As Zhang et al. (2021) point out, the developmental features of lexical density varies with learners' proficiency level. It is also possible to explain the discrepancy through the degree of exposure to lexical words. Specifically, a major focus of vocabulary teaching is functional words in learning stage 1, whereas extensive instruction in lexical words is offered from learning stage 2 to 3. Therefore, L3 beginner learners' lexical density develops across three learning stages.

Moreover, the upper learning stages no longer exclusively use nouns but rather begin to use a wide range of lexical word classes, including verbs, adjectives, and adverbs. According to the DUB approach, sufficient linguistic input could accelerate the lexical learning process. Therefore, this apparent increase in various word classes in the upper learning stages may also be explained by extensive exposure to lexical words.

## Conclusion

This cross-sectional study investigated the developmental features of lexical richness in L3 writing samples by Chinese L3 beginner learners from the perspective of the DUB. The results indicate that lexical richness in L3 beginner learners' writing is generally low. Specifically, L3 beginner learners use fewer diverse words and lexical words but use numerous high-frequency words in their writing, which indicates that L3 beginner learners' writing is characterized by informal and spoken-like register. Additionally, from the developmental perspective, lexical sophistication and lexical density yield positive growth across the three learning stages, but lexical diversity develops non-linearly.

The present study makes several noteworthy contributions with respect to lexical richness in L3 writings by L3 beginner learners. First, these findings enhance our understanding of the developmental features of lexical richness. In particular, earlier studies identified a lexical plateau among college-level students (Zhu and Wang, 2013; Wang and Wang, 2020), while the present study suggests that the lexical plateau appears earlier in third language acquisition. Second, the present study confirms Zhang et al.'s (2021) finding that sufficient exposure to words may be a critical condition for the development of lexical richness. This finding suggests that linguistic exposure plays a significant role in promoting lexical richness. Finally, this study utilizes a new word list that is suitable for beginner learners. On the one hand, it can verify the usages of new word lists. On the other hand, we expect that these findings will be useful in helping teachers to understand the developmental features of lexical richness in L3 writing by L3 beginner learners. In summary, the present study has explored the multidimensional development of lexical richness in Chinese L3 beginner learners' writing, which will be useful for teachers and learners to indentify the developmental features of lexical richness in L3 writing. In addition, the study provides a better understanding of the dynamic development of L3 writing, which also enriches the literature based on the DUB approach in applied linguistics.

These findings have several implications for L3 writing research and vocabulary teaching. First, teachers should provide more low-frequency words to enhance learners' lexical sophistication. Second, since L3 beginner learners tend to use less diverse words to express the same ideas in the text, numerous various and sophisticated words could be presented in multiple contexts. Third, owing to their spoken-like writing style, teachers should help learners better understand the differences in lexical features between spoken and written tasks by analyzing the model texts. Fourth, teachers should provide more exercises to encounter different lexical word classes in other contexts to help L3 beginner learners use diverse lexical word classes. Finally, in view of the complex competition between language systems and the lack of available resources in third language acquisition, besides offering more extensive exposure to L3 vocabulary, teachers should also help L3 beginner learners to improve their internal factors for L3 learning, such as learning motivation.

However, it is important to note three limitations of this study. First, the sample size of the corpus is relatively small. The small size may have some effects on our findings. Second, the present study adopts the cross-sectional data to investigate the developmental features of lexical richness across the three learning stages. It is not possible to observe how lexical richness develops over time in L3 writing. Finally, since learning context plays a critical role in language learning, this study, which focuses exclusively on L3 beginner learners of English in Chinese contexts, may be insufficient to determine whether our findings represent universal developmental features of lexical richness in L3 writing or are specific to Chinese L3 beginner learners of English. Future studies should use a longitudinal design and expand the sample size of corpus and writing samples by L3 beginner learners of English in other learning contexts to more comprehensively investigate the developmental features of lexical richness in L3 writing.

## Data Availability Statement

The original contributions generated for this study are included in the article/Supplementary Material, further inquiries can be directed to the corresponding author.

## Ethics Statement

The studies involving human participants were reviewed and approved by Professor Committee of School of Foreign Languages, Northeast Normal University. Written informed consent from the participants' legal guardian/next of kin was not required to participate in this study in accordance with the national legislation and the institutional requirements.

## Author Contributions

XL was responsible for the conception and design of the study, data collection, data analysis and interpretation, writing, developing, and editing the manuscript. HZ contributed to the conception and design of the study, data analysis and interpretation, manuscript development, and the editing. All authors contributed to the article and approved the submitted version.

## Funding

This research was funded by National Office for Philosophy and Social Sciences of China (Fund No. 20BYY209).

## Conflict of Interest

The authors declare that the research was conducted in the absence of any commercial or financial relationships that could be construed as a potential conflict of interest.

## Publisher's Note

All claims expressed in this article are solely those of the authors and do not necessarily represent those of their affiliated organizations, or those of the publisher, the editors and the reviewers. Any product that may be evaluated in this article, or claim that may be made by its manufacturer, is not guaranteed or endorsed by the publisher.
