# Supplementary material for: Developmental Features of Lexical Richness in English Writings by Chinese L3 Beginner Learners
Source: Front Psychol. 2021 Sep 24;12:752950. doi: 10.3389/fpsyg.2021.752950 (PMC8498328; doi:10.3389/fpsyg.2021.752950)
Supplement: Supplementary file 1 [file Data_Sheet_1.PDF]

Appendix. Students' Writing Samples

1. Student X in learning stage 1
Self-introduction

Dear Sir,

My name is Li Hua. My first name is Hua and my last name is Li. I am 12 years old. I am a No. 5 Middle School student. I like English, playing volleyball and computer games. I can draw, play the guitar and sing English songs. And my habit is tell English stories. It's very interesting. This is me. How about you? Please write me soon.

Yours,

Li Hua

2. Student X in learning stage 2
Introduction to career planning

Dear teachers and classmates:

I'm Li Hua. This is my report. My dream is want to be a teacher when I grow up. At first, as a teacher, you should learn more knowledge and teach students in this ways. My advantage is I often listen to teachers carefully and write down notebooks. On the other hand, I often ask questions at the free time. So I will learn to study hard and do lots of exercise at home. I believe I will achieve my dream.

Yours,

Li Hua

3. Student X in learning stage 3
Introduction to traditional festivals

Dear Lucy,

How are you recently? The Double Ninth Festival is coming. It is very meaningful. It means we should company the old. So would you like to go with me to do some meaningful things?

My classmates and I plan to go nursing home which has many lonely old people. We will meet at 9:00 in front of the school gate. We will sing and dance to make them happy. In addition, we will make dumplings together. We will go back at 5:00 pm. What a happy day!

I do hope you could go with us. It not only can bring them happiness but also is very meaningful. I am looking forward to receiving your reply.

Yours,

Li Hua
